# Integrated Omic Analysis of Human Plasma Metabolites and Microbiota in a Hypertension Cohort

**DOI:** 10.3390/nu15092074

**Published:** 2023-04-25

**Authors:** Bo-Yan Chen, Yu-Lin Li, Wen-Zhen Lin, Chao Bi, Lin-Juan Du, Yuan Liu, Lu-Jun Zhou, Ting Liu, Shuo Xu, Jun Zhang, Yan Liu, Hong Zhu, Wu-Chang Zhang, Zhi-Yuan Zhang, Sheng-Zhong Duan

**Affiliations:** 1 Department of Oral and Maxillofacial-Head and Neck Oncology, Shanghai Ninth People’s Hospital, Shanghai Jiao Tong University School of Medicine, Shanghai 200011, China; chenboyananderson@foxmail.com; 2 Laboratory of Oral Microbiota and Systemic Diseases, Shanghai Ninth People’s Hospital, College of Stomatology, Shanghai Jiao Tong University School of Medicine, Shanghai 200125, China; liyulin597926717@sjtu.edu.cn (Y.-L.L.); janetist@outlook.com (W.-Z.L.); dulinjuan@sibs.ac.cn (L.-J.D.); liuyuan@sibs.ac.cn (Y.L.); lujun_zhou@163.com (L.-J.Z.); emmaliu123@126.com (T.L.); xushuo0177@163.com (S.X.); hamiltonz@163.com (J.Z.); zibao2005@hotmail.com (Y.L.); zhuhongmed@126.com (H.Z.); zhangwuchang104@shsmu.edu.cn (W.-C.Z.); 3 National Center for Stomatology; National Clinical Research Center for Oral Diseases; Shanghai Key Laboratory of Stomatology, Shanghai 200125, China; 4 Department of Stomatology, First Affiliated Hospital, Anhui Medical University, Hefei 230022, China; bchao1025@163.com; 5 Department of General Dentistry, Shanghai Ninth People’s Hospital, Shanghai Jiao Tong University School of Medicine, Shanghai 200011, China

**Keywords:** oral microbiota, gut microbiota, plasma metabolites, hypertension

## Abstract

Hypertension is closely related to metabolic dysregulation, which is associated with microbial dysbiosis and altered host–microbiota interactions. However, plasma metabolite profiles and their relationships to oral/gut microbiota in hypertension have not been evaluated in depth. Plasma, saliva, subgingival plaques, and feces were collected from 52 hypertensive participants and 24 healthy controls in a cross-sectional cohort. Untargeted metabolomic profiling of plasma was performed using high-performance liquid chromatography–mass spectrometry. Microbial profiling of oral and gut samples was determined via 16S rRNA and metagenomic sequencing. Correlations between metabolites and clinic parameters/microbiota were identified using Spearman’s correlation analysis. Metabolomic evaluation showed distinct clusters of metabolites in plasma between hypertensive participants and control participants. Hypertensive participants had six significantly increased and thirty-seven significantly decreased plasma metabolites compared to controls. The plasma metabolic similarity significantly correlated with the community similarity of microbiota. Both oral and gut microbial community composition had significant correlations with metabolites such as Sphingosine 1-phosphate, a molecule involved in the regulation of blood pressure. Plasma metabolites had a larger number of significant correlations with bacterial genera than fungal genera. The shared oral/gut bacterial genera had more correlations with metabolites than unique genera but shared fungal genera and metabolites did not show clear clusters. The hypertension group had fewer correlations between plasma metabolites and bacteria/fungi than controls at species level. The integrative analysis of plasma metabolome and oral/gut microbiome identified unreported alterations of plasma metabolites in hypertension and revealed correlations between altered metabolites and oral/gut microbiota. These observations suggested metabolites and microbiota may become valuable targets for therapeutic and preventive interventions of hypertension.

## 1. Introduction

Hypertension (HTN) is a complex disease with a multitude of symptoms and etiologies associated with many organs. Accumulating interest has focused on the role of the microbiota in HTN [1,2]. The associations between microbiota and many diseases can be attributed to metabolites of the microbiota that enter the circulation [3]. Even though animal experiments have demonstrated that metabolic signaling may explain the mechanism between microbiota and blood pressure [4,5], the characteristics of blood metabolites and their relationships with microbiota in the HTN population remain to be further explored. Therefore, it is of significant interest to reveal the alterations of plasma metabolites and the influence of microbiota on metabolites in HTN in order to comprehend possible etiological factors and potential new therapeutic strategies for HTN.

Plasma metabolome is an important approach to identify metabolic biomarkers and causative agents of various diseases. Recent studies have discovered that metabolites in peripheral blood are associated with the development of liver disease [6], arthritis [7], chronic kidney disease [8], and Alzheimer’s disease [9]. In patients with HTN, altered serum metabolic signatures may involve metabolism of fatty acid, glycerophospholipid, alanine, aspartate, and glutamate [10,11]. In addition, significantly different metabolites in HTN have been identified in a pilot study and a randomized clinical trial [12,13]. Some metabolites are known to originate from or are influenced by the oral and gut microbiota [14,15]. For example, gut microbiota-derived short-chain fatty acids and trimethylamine *N*-oxide exert beneficial and detrimental effects on HTN, respectively [16,17]. Nitrate-responsive oral microbiota modulates blood pressure in both human subjects and animal models [18,19]. Pathophysiological alterations of the oral cavity and intestine create preconditions for metabolites to enter the circulation [20,21]. These studies emphasize the important role of metabolites in HTN and suggest that microbial dysbiosis affects the development and progression of HTN at least partially through microbial metabolites. Although some studies have observed aberrant blood metabolic patterns in the pathogenesis of HTN [1], the relationship between metabolites and human microbiota has not been well characterized, particularly concerning oral/gut fungi.

In the current study, we revealed plasma metabolic features of HTN by non-targeted metabolomics analysis and established their correlations with oral/gut bacteria and fungi. We elucidated significantly altered plasma metabolites in HTN. We further depicted associations between metabolite and bacteria/fungi at the species and genus level.

## 2. Results

### 2.1. Metabolomic Profile of Fasting Plasma from No HTN and HTN Participants

**Figure 1:**
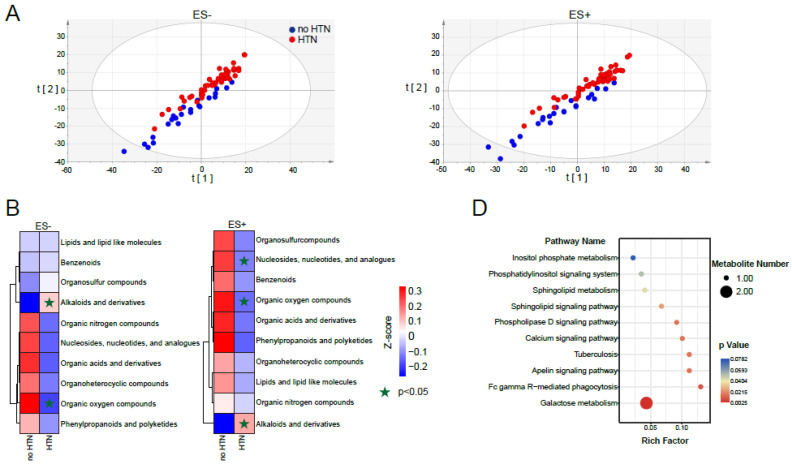

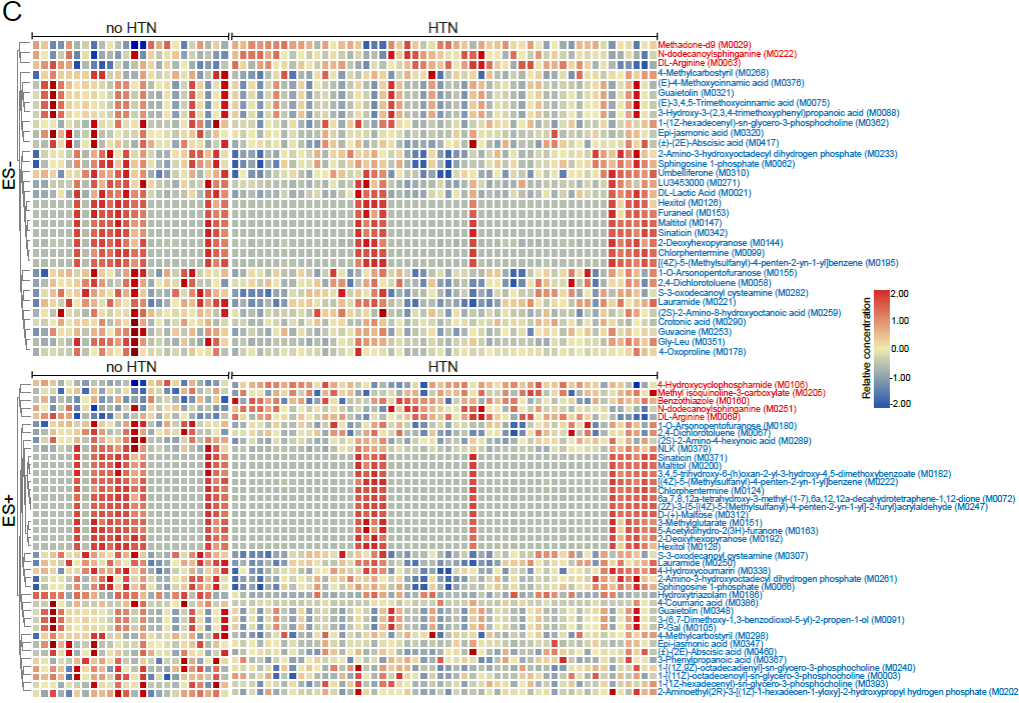
Alterations of fasting plasma metabolites in HTN participants. (**A**). PLS-DA score plots of plasma metabolic profiles of participants with or without HTN in ES− or ES+. ES−: negative ion mode; ES+: positive ion mode. (**B**). Clustering heatmaps showing the differences in superclass of plasma metabolites between no HTN and HTN. Plasma metabolites were matched in the Human Metabolome Database (HMDB). The expressions of the metabolic superclass were transformed into Z scores by subtracting the average values and dividing the standard deviation of all samples. (**C**). Clustering heatmaps of plasma metabolites were significantly different between no HTN and HTN (*p* < 0.05, VIP > 1) in ES− and ES+. Blue and red text represent no HTN- and HTN-enriched metabolites, respectively. (**D**). Bubble chart of significantly different KEGG metabolic pathways (based on plasma metabolites) between no HTN and HTN. Rich factor equals the number of differential metabolites enriched in a pathway divided by the number of all metabolites enriched in this pathway. n = 24 vs. 52 for no HTN vs. HTN.

To compare the blood metabolic characteristics between no HTN and HTN participants, we profiled fasting plasma metabolites using non-targeted metabolomics. Demographic characteristics of the cohort are summarized in Table 1. Partial least squares discrimination analysis (PLS-DA) and Principal Component Analysis (PCA) succeed in distinguishing samples of plasma metabolomics between no HTN and HTN (Figure 1A and Figure S1). For both ES− (negative ion mode) and ES+ (positive ion mode), the superclass alkaloids and derivatives were significantly enriched in HTN, and organic oxygen compounds were significantly enriched in no HTN (Figure 1B). Nucleosides, nucleotides, and analogues were significantly enriched in the no HTN group only in ES+ (Figure 1B). Comparisons of the relative concentrations of these metabolites revealed that HTN had six significantly increased and thirty-seven significantly decreased plasma metabolites compared to no HTN (Figure 1C). Specifically, three metabolites significantly increased and twenty-nine significantly decreased in participants with HTN versus no HTN in ES−, and five significantly increased and thirty-four significantly decreased in ES+ (Figure 1C). Galactose metabolism was the most enriched pathway of the significantly changed metabolites annotated by KEGG (Figure 1D).

### 2.2. Correlations between Plasma Metabolites and Clinical Parameters

**Figure 2:**
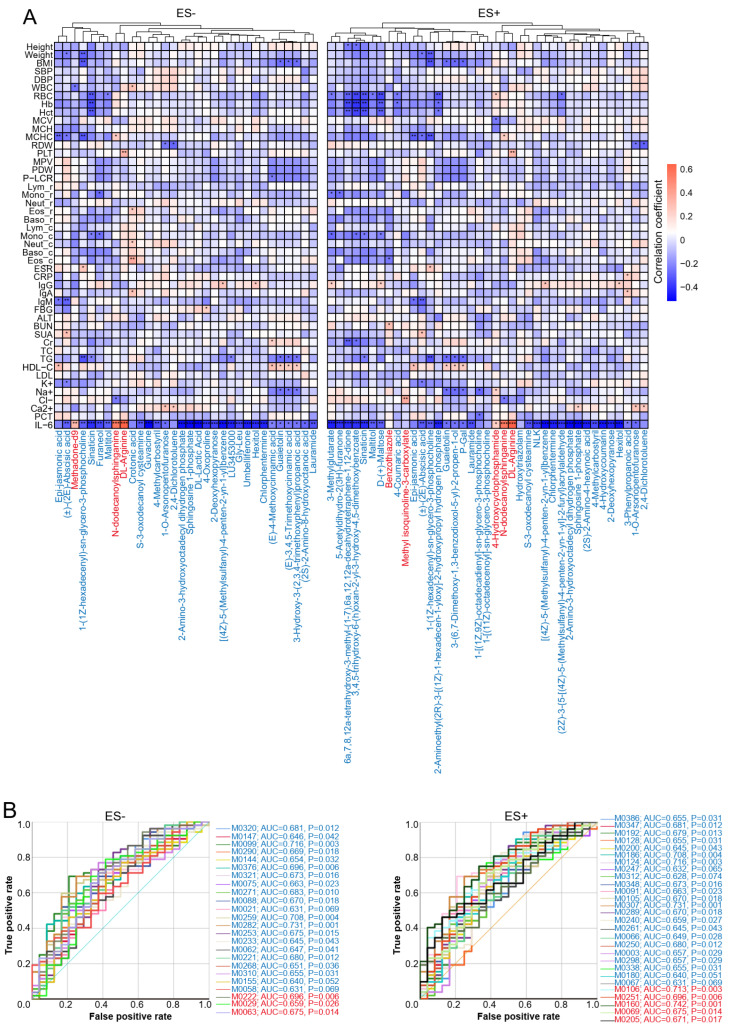
Associations between altered metabolites and clinical parameters. (**A**). Clustering heatmaps of Spearman’s correlation coefficients between clinical parameters and metabolites differentially enriched in no HTN and HTN. BMI: body mass index, SBP: systolic blood pressure, DBP: diastolic blood pressure, ESR: erythrocyte sedimentation rate, RBC: red blood cells, Hb: hemoglobin, Hct: hematocrit, MCV: mean corpuscular volume, MCH: mean corpuscular hemoglobin, MCHC: mean corpuscular hemoglobin concentration, RDW: red blood cell distribution width, PLT: platelet, MPV: mean platelet volume, PDW: platelet distribution width, P-LCR: platelet-large cell ratio, Ly_ r: Lymphocyte ratio, Mono_r: Monocyte ratio, Neut_ r: Neutrophil ratio, Eo_r: Eosinophil ratio, Baso_ r: Basophil ratio, Ly_c: Lymphocyte count, Mono_c: Monocyte count, Neut_c: Neutrophil count, Baso_c: Basophil count, Eo_c: Eosinophil count, WBC: White blood cells, Ig: Immunoglobulin, ALT: alanine aminotransferase, BUN: blood urea nitrogen, SUA: serum uric acid, Cr: creatinine, FBG: fasting blood glucose, TC: total cholesterol, TG: triglyceride, HDL-C: high density lipoprotein cholesterol, LDL: low-density lipoprotein cholesterol, K+: potassium, Na+: sodium, Cl-: chlorine, Ca2+: calcium, CRP: C-reactive protein, PCT: Procalcitonin, IL-6: Interleukin-6. (**B**). ROC analysis for prediction of HTN using significantly different plasma metabolites between no HTN and HTN in ES− and ES+. Blue and red text represent no HTN- and HTN-enriched metabolites, respectively. n = 24 vs. 52 for no HTN vs. HTN. * *p*(FDR) < 0.05, ** *p*(FDR) < 0.01, *** *p*(FDR) < 0.001.

The concentration of plasma metabolites is associated with different clinical parameters in various diseases [7,22]. To analyze whether any of the clinical data have interactions with altered plasma metabolites, we carried out Spearman’s correlation analysis between 47 clinical parameters and the concentration of plasma metabolites. We detected 34 parameters significantly correlated with plasma metabolites (Figure 2A). Most of these correlations were negative, including 59 negative and 23 positive associations in ES− and 85 negative and 24 positive associations in ES+ (Figure 2A).

Although plasma metabolites did not directly correlate with either SBP or DBP, significant correlations were detected between plasma metabolites and BP-related clinical indicators (Figure 2A). Levels of plasma triglyceride (TG) and high-density lipoprotein-cholesterol (HDL−C) are closely associated with HTN [23,24,25]. We identified that TG negatively correlated with six no HTN-enriched metabolites in ES− and five in ES+, and HDL-C positively correlated with five no HTN-enriched metabolites in ES− and four in ES+ (Figure 2A). Interestingly, five metabolites negatively correlated with TG and positively correlated with HDL-C at the same time, including (E)-3,4,5-Trimethoxycinnamic acid (M0075) and 3-Hydroxy-3-(2,3,4-trimethoxyphenyl) propanoic acid (M0088) in ES−; 3-(6,7-Dimethoxy-1,3-benzodioxol-5-yl)-2-propen-1-ol (M0091) and P-Gal (M0105) in ES+; and guaietolin (M0321) in ES− and ES+ (Figure 2A). Both high sodium intake and overweight are risk factors for HTN [26,27,28,29]. The above-mentioned five no HTN-enriched metabolites also negatively correlated with plasma sodium and BMI in ES− and ES+ (Figure 2A). Among inflammatory markers, IL-6 significantly correlated almost all altered metabolites (Figure 2A). Intriguingly, the correlations were negative between IL-6 and no HTN-enriched metabolites and positive between IL-6 and HTN-enriched metabolites (Figure 2A). These results suggested that plasma metabolites affected BP in multiple ways indirectly.

Given the substantial changes of metabolites in HTN and the notable correlations between clinical parameters and metabolic signatures, we further assessed the potential value of using metabolites to discriminate HTN. Receiver operating characteristic (ROC) curves of the 24 significantly altered metabolites in ES− and the 27 in ES+ yielded an area under the curve (AUC) higher than 60% (Figure 2B, Tables S1 and S2), suggesting that these metabolites had a classifying ability to discriminate participants with HTN from no HTN. Metabolites with an AUC value over 70%, including S-3-oxodecanoyl cysteamine and Chlorphentermine in ES− (M0282 and M0099) and ES+ (M0307 and M0124), Hydroxytriazolam (M0186) and 4-Hydroxycyclophosphamide (M0106) in ES+, and (2S)-2-Amino-8-hydroxyoctanoic acid (M0259) in ES−, presented enough sensitivity and specificity to differentiate no HTN from HTN (Figure 2B). ROC curve of combined metabolites of ES (+) enriched in no HTN had the highest AUC value (Figure S2).

### 2.3. Associations between Plasma Metabolites and Community Composition of Oral and Gut Microbes

**Figure 3:**
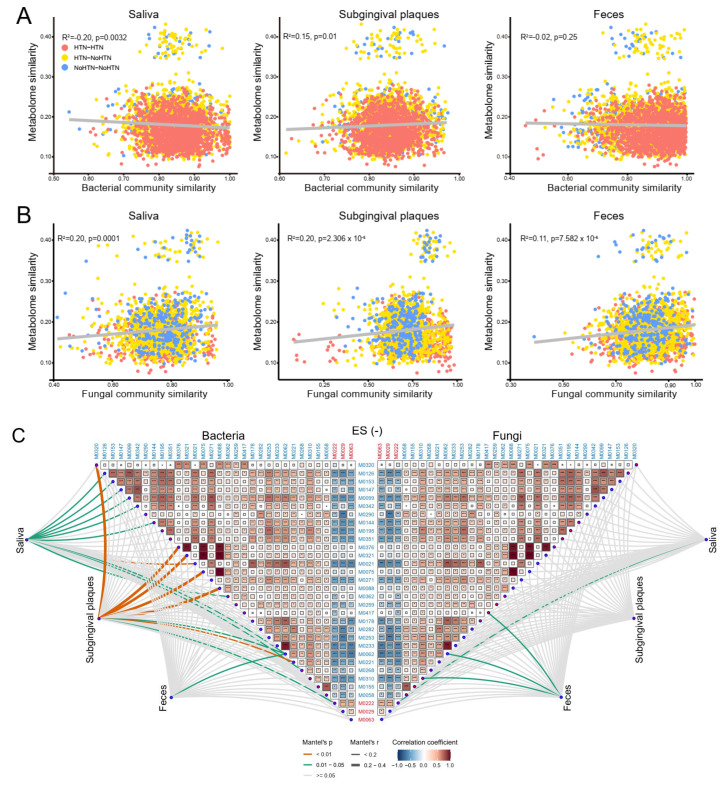

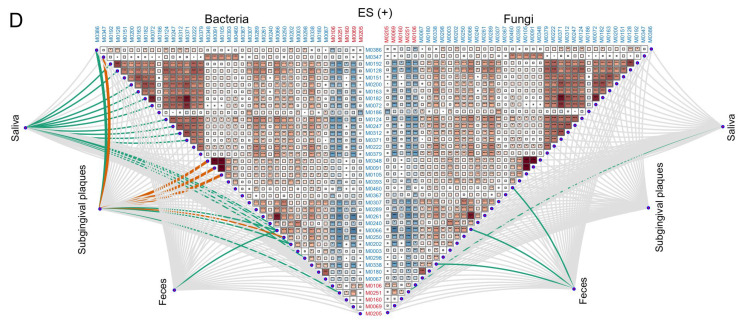
Associations between plasma metabolites and community composition of oral and gut microbes. (**A**,**B**). Correlations between bacterial (**A**) or fungal (**B**) community similarity and metabolome similarity. All similarities were calculated based on the Bray–Curtis distance, and similarity for microbiome was calculated at the species level. (**C**,**D**). Correlation analysis within altered metabolites as well as between altered metabolites and microbial community composition in ES− (**C**) or ES+ (**D**). Color gradient denotes Spearman’s correlation coefficient between different metabolites. Bacterial and fungal community composition of saliva, subgingival plaques, and feces were related to each altered metabolite via partial Mantel tests. Edge width represents the Mantel’s r statistic for the correlations, and edge color denotes statistical significance. Blue and red text represent no HTN- and HTN-enriched metabolites, respectively. For bacteria-related analysis, 16S rRNA gene sequencing was used, and metagenomic sequencing was used for fungi-related analysis. n = 76 for all samples. * *p*(FDR) < 0.05, ** *p*(FDR) < 0.01, *** *p*(FDR) < 0.001.

The oral and gut microbiota have been demonstrated to influence HTN and blood metabolome [18,30,31,32]. Our previous works have investigated the diversity and composition of bacterial and fungal microbes in both oral and gut samples [33,34]. To understand the associations between microbiota and host metabolites in HTN, we first analyzed correlations of similarities between metabolome and microbial community. We observed that plasma metabolic similarity significantly correlated with the bacterial and fungal community similarity (Figure 3A,B). Plasma metabolic similarity negatively correlated with bacterial community similarity of saliva, and positively correlated with that of subgingival plaques, but did not significantly correlate with that of feces (Figure 3A). All associations between plasma metabolic similarity and fungal community similarity of the oral cavity and the gut were positive (Figure 3B). We also analyzed relationships between different metabolites in ES− and ES+ (Figure 3C,D). Correlations within metabolites enriched in no HTN were all positive, as were those within metabolites enriched in HTN (Figure 3C,D). In contrast, correlations between no HTN-enriched and HTN-enriched metabolites were all negative (Figure 3C,D).

To explore the detailed associations between metabolites and the overall microbial community, we performed a partial Mantel test between the concentration of plasma metabolites and community composition of oral and gut microbiota at the phylum level (Figure 3C,D). Overall, there were larger number of significant correlations between metabolites and bacterial community composition than between metabolites and fungal community composition in both ES− and ES+ (Figure 3C,D). There were a larger number of significant correlations between metabolites and bacterial community composition in the oral cavity than the gut, whereas there were a larger number of significant correlations between metabolites and fungal community composition in the gut than the oral cavity (Figure 3C,D). Epi-jasmonic acid (M0320 in ES− and M0347 in ES+) and guaietolin (M0321 in ES− and M0348 in ES+); (E)-4-Methoxycinnamic acid (M0376), M0075, and M0088 in ES−; and 3-(6,7-Dimethoxy-1,3-benzodioxol-5-yl)-2-propen-1-ol (M0091) and M0105 in ES+ strongly correlated with the bacterial community composition in subgingival plaques (Figure 3C,D). For HTN-enriched metabolites, only *N*-dodecanoylsphinganine (M0222 in ES− and M0251 in ES+) significantly correlated with bacterial community composition of subgingival plaques and fungal community composition of saliva (Figure 3C,D). Sphingosine 1-phosphate (S1P) is a pivotal regulator of BP, as previously reported [35,36]. This metabolite caught our attention because it significantly correlated with the bacterial community composition of saliva, subgingival plaques, and feces in both ES− (M0062) and ES+ (M0066) (Figure 3C,D). The fungal community composition of feces but not saliva or subgingival plaques significantly correlated with S1P in ES− and ES+ (Figure 3C,D). These results suggested critical interactions between microbial community composition and plasma metabolites and highlighted a possible pattern of microbiota influencing BP through metabolites.

### 2.4. Associations between Plasma Metabolites and Oral/Gut Bacteria at Genus Level

**Figure 4:**
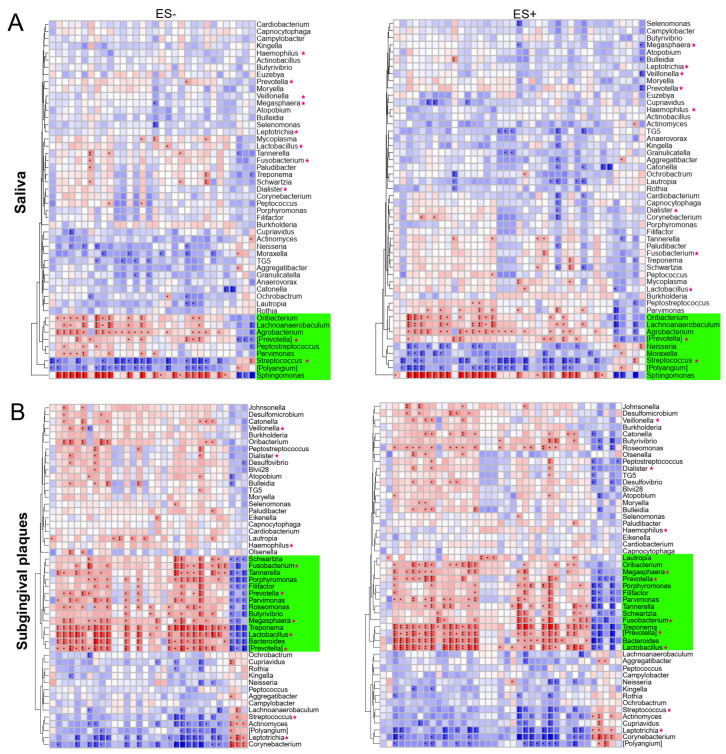

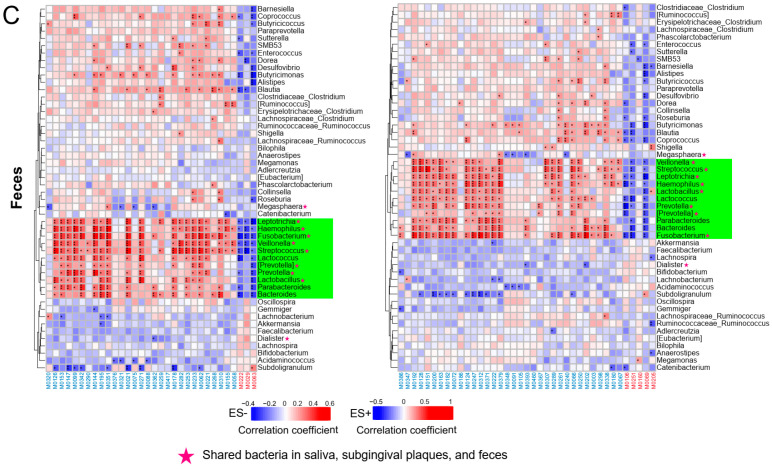
Associations between oral/gut bacterial genera and fasting plasma metabolites. Clustering heatmaps of Spearman’s correlation coefficients between plasma metabolites in ES− or ES+ and the top 50 bacterial genera in saliva (**A**), subgingival plaques (**B**), and feces (**C**). n = 76 for saliva and feces, and 75 for subgingival plaques. Blue and red text represent no HTN- and HTN-enriched metabolites, respectively. For bacteria-related analysis, 16S rRNA gene sequencing was used at genus level. * *p*(FDR) < 0.05, ** *p*(FDR) < 0.01, *** *p*(FDR) < 0.001.

To further investigate the relationships between plasma metabolites and microbiota in HTN, we analyzed the correlations between metabolites and the relative abundance of the top 50 oral/gut bacterial genera. In ES−, the 3 HTN-enriched metabolites had 20 significant positive correlations and 90 significant negative correlations with the oral/gut bacterial genera, and the 29 no HTN-enriched metabolites had 99 significant positive correlations and 56 significant negative correlations with bacterial genera (Figure 4A–C). The trend of correlations between microbiota and the altered metabolites in ES+ was similar to that in ES− (Figure 4A–C). These results revealed a larger number of significant positive correlations than negative correlations between no HTN-enriched metabolites and bacterial genera in saliva, subgingival plaques, and feces, whereas the opposite was true between HTN-enriched metabolites and the same genera.

Correlation heatmaps clustered the microbiota that had similar correlative patterns with altered metabolites (Figure 4A–C). For example, nine salivary genera, fourteen subgingival genera, and eleven gut genera had significant correlations with plasma metabolites in ES− and ES+ and were clustered (Figure 4A–C, highlighted in green). Comparing the top 50 genera of the salivary, subgingival, and gut microbiota identified a total of 10 shared genera, including *Megasphaera*, *Dialister*, *Streptococcus*, *Haemophilus*, *Lactobacillus*, *Leptotrichia*, *Veillonella*, *[Prevotella]*, *Fusobacterium*, and *Prevotella* (Figure 4A–C, marked by stars). Interestingly, the number of shared genera increased from the oral cavity to the gut in the green-highlighted clusters (*Streptococcus* and *[Prevotella]* in saliva; *Fusobacterium*, *Prevotella*, *Megasphaera*, *Lactobacillus* and *[Prevotella]* in subgingival plaques; and *Streptococcus*, *Haemophilus*, *Lactobacillus*, *Leptotrichia*, *Veillonella*, *[Prevotella]*, *Fusobacterium* and *Prevotella* in gut; Figure 4A–C). Of the green-highlighted shared genera in ES− and ES+, except for salivary *Streptococcus* and gut *Lactobacillus*, the significant correlations between these bacterial genera and no HTN-enriched metabolites were all positive, while the correlations between these genera and HTN-enriched metabolites were all negative (Figure 4A–C). Intriguingly, except for subgingival *Megasphaera* and *Prevotella* in ES+, the green-highlighted shared genera in saliva, subgingival plaques, and feces significantly correlated with S1P (M0062 in ES− and M0066 in ES+) (Figure 4A–C). Furthermore, except for *Megasphaera* and *Dialister*, the rest of the shared genera (8 of 10) clustered together in the gut and showed exceedingly significant correlations with plasma metabolites, especially Furaneol (M0153), Maltitol (M0147), Gly−Leu (M0351), S1P, and DL-Arginine (M0063), each of which significantly correlated with all eight shared genera (Figure 4C). These results further demonstrated close relationships between shared oral/gut microbiota and plasma metabolites.

### 2.5. Associations between Plasma Metabolites and Oral/Gut Fungi at Genus Level

**Figure 5:**
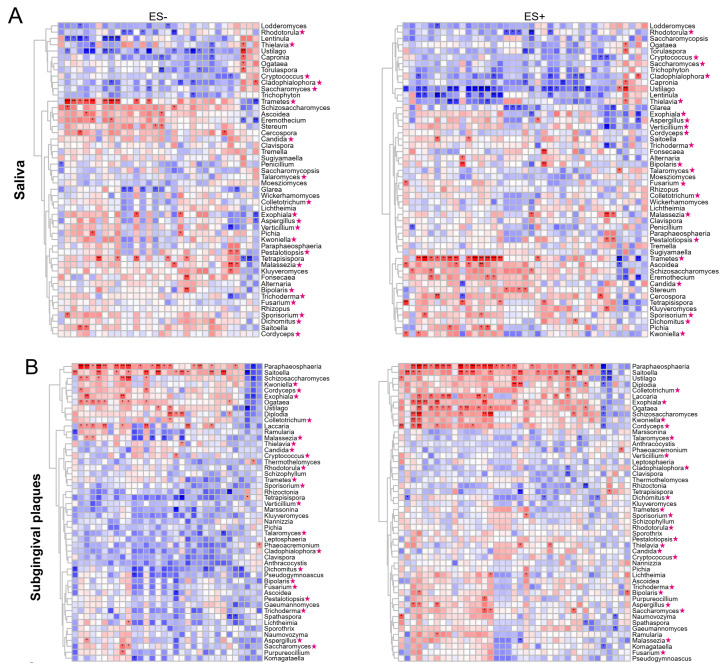

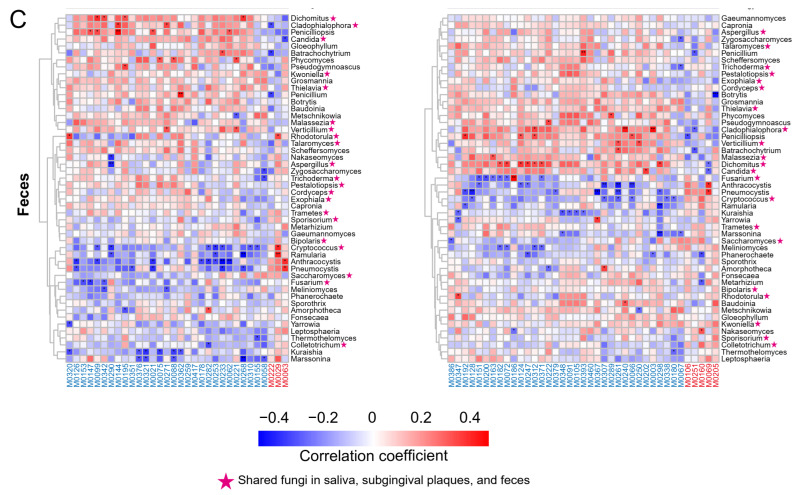
Associations between oral/gut fungal genera and fasting plasma metabolites. Clustering heatmaps of Spearman’s correlation coefficients between plasma metabolites in ES− or ES+ and the top 50 fungal genera in saliva (**A**), subgingival plaques (**B**), and feces (**C**). n = 60 for all sample types. Blue and red text represent no HTN- and HTN-enriched metabolites, respectively. Metagenomic sequencing was used for fungi-related analysis at genus level. * *p*(FDR) < 0.05, ** *p*(FDR) < 0.01, *** *p*(FDR) < 0.001.

We also analyzed the correlations between metabolites and the relative abundance of the top 50 oral/gut fungal genera (Figure 5). We identified 14 significant positive correlations and 16 significant negative correlations between fungal genera and HTN-enriched metabolites, as well as 132 significant positive correlations and 111 significant negative correlations between fungal genera and no HTN-enriched metabolites in ES− (Figure 5A–C). Similar results were observed in ES+ (Figure 5A–C). Consistent with the relationships between bacteria and metabolites, correlations between HTN-enriched metabolites and fungal genera were usually the opposite to associations between no HNT-enriched metabolites and these genera (Figure 5A–C). *Trametes*, *Paraphaeosphaeria*, and *Dichomitus* had the largest number of significant positive correlations with metabolites in saliva, subgingival plaques, and feces, respectively, and the fungal genera had the largest number of significant negative correlations with metabolites and were salivary *Ustilago*, subgingival *Dichomitus*, and gut *Anthracocystis* (Figure 5A–C). Unlike the correlations between bacterial genera and metabolites, those between fungal genera and metabolites did not show clear clusters (Figure 5A–C). We also identified 21 fungal genera shared in the oral cavity and the gut (Figure 5A–C, marked by stars). These results established the correlations between fungal genera and HTN-related metabolites (Figure 5A–C).

### 2.6. Associations between Plasma Metabolites and Oral/Gut Microbiota at the Species Level

**Figure 6:**
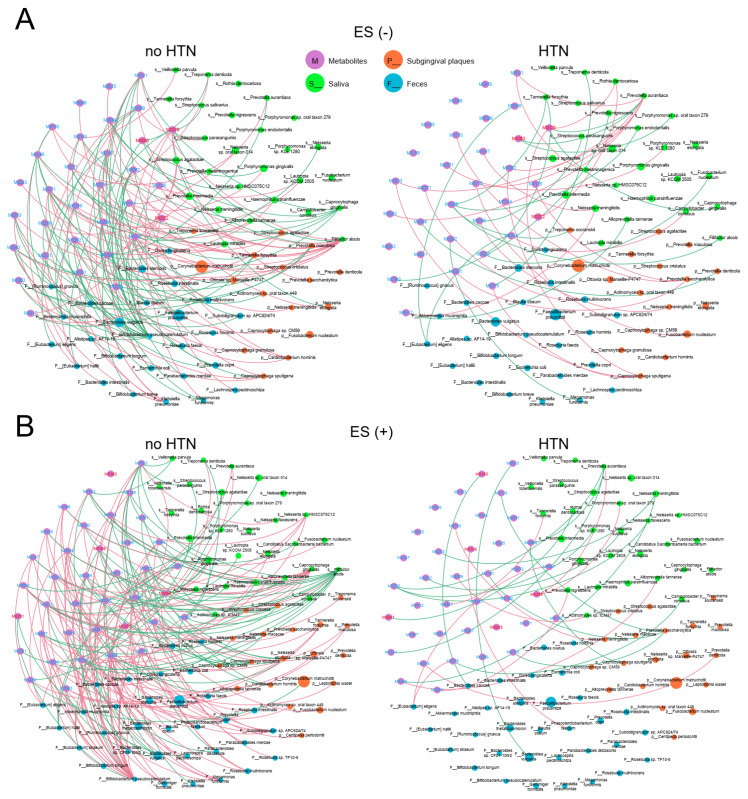
Associations between oral/gut bacterial species and fasting plasma metabolites. Differentially enriched metabolites of the no HTN and HTN groups were analyzed for correlations with the bacterial abundance of saliva, subgingival plaques, and feces in ES− (**A**) and ES+ (**B**). The size of circles indicates the relative abundance of each species. Pink and green lines indicate positive and negative correlations, respectively. The top 50 most abundant species in saliva, subgingival plaques, and feces were used for Spearman’s correlation analysis, and correlations with r > 0.45 and *p* < 0.05 were displayed. Metagenomic sequencing was used for bacteria-related analysis at species level. n = 24:36 (no HTN vs. HTN) for all sample types.

**Figure 7:**
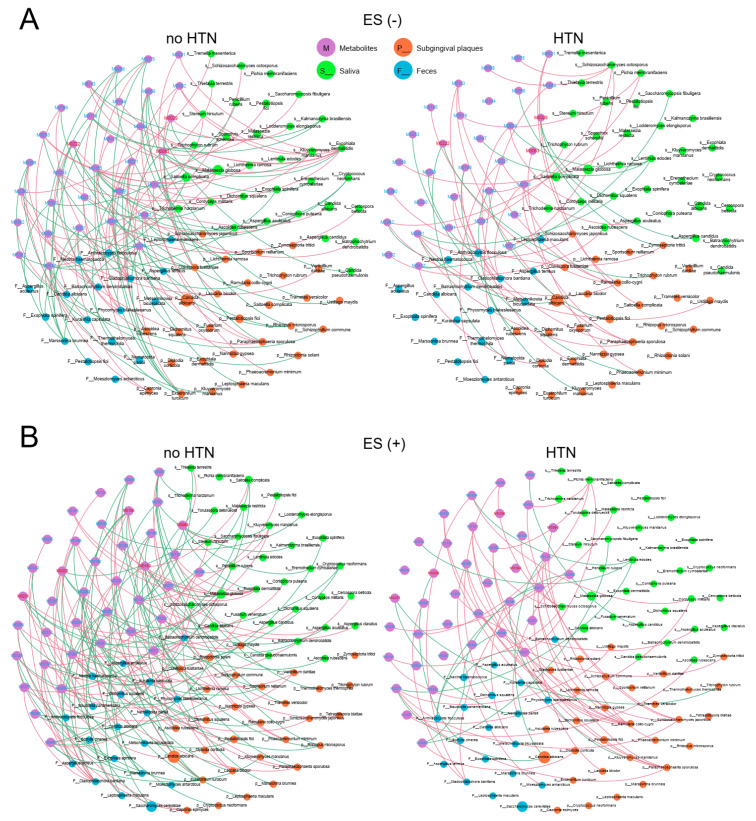
Associations between oral/gut fungal species and fasting plasma metabolites. Differentially enriched metabolites of the no HTN and HTN groups were analyzed for correlations with the fungal abundance of saliva, subgingival plaques, and feces in ES− (**A**) and ES+ (**B**). The size of circles indicates the relative abundance of each species. Pink and green lines indicate positive and negative correlations, respectively. The top 50 most abundant species in saliva, subgingival plaques, and feces were used for Spearman’s correlation analysis, and correlations with r > 0.45 and *p* < 0.05 were displayed. Metagenomic sequencing was used for fungi-related analysis at species level. n = 24:36 (no HTN vs. HTN) for all sample types.

To understand the potential interplay among differentially enriched plasma metabolites and microbiota in the no HTN and HTN groups, we performed Spearman’s correlation analysis using microbial species and constructed ecological networks (Figure 6 and Figure 7). Strikingly, the the no HTN and HTN networks between the top 50 microbial species and metabolites were dramatically different. In the no HTN group, we identified 133 significant correlations between metabolites and bacteria in ES− (Figure 6A) and 174 in ES+ (Figure 6B), as well as 117 significant correlations between metabolites and fungi in ES− (Figure 7A) and 138 in ES+ (Figure 7B). In the HTN group, there were 40 significant correlations between metabolites and bacteria in ES− (Figure 6A) and 27 in ES+ (Figure 6B), as well as 50 significant correlations between metabolites and fungi in ES− (Figure 7A) and 58 in ES+ (Figure 7B). These results suggested that correlations between oral/gut microbiota and plasma metabolites were impaired in the HTN group (Table S3).

We further analyzed the ecological networks between bacteria and metabolites as well as between fungi and metabolites in detail. In the no HTN group, there were 15 negative and 29 positive correlations in saliva, 11 negative and 15 positive correlations in subgingival plaques, and 46 negative and 17 positive correlations in feces between bacterial species and metabolites in ES− (Figure 6A). Similarly, we observed a larger number of positive correlations than negative ones in the oral cavity and a larger number of negative correlations than positive ones in the gut of the no HTN group in ES+ (Figure 6B). These results revealed that gut bacteria from the no HTN group had more associations with plasma metabolites than oral bacteria (Figure 6A,B). However, these associations between metabolites and bacterial species were more enriched in saliva when compared with the subgingival plaques and feces of the HTN group in both ES− and ES+ (Figure 6A,B). Notably, gut *Bifidobacterium pseudocatenulatum* had the largest number of significant correlations with metabolites among the top 50 bacterial species in the no HTN group, and salivary *Prevotella aurantiaca* had the largest number of significant correlations with metabolites in the HTN group (Figure 6A,B).

For associations between fungal species and metabolites in the no HTN group, we found 18 negative and 25 positive correlations for saliva, 25 negative and 14 positive correlations for subgingival plaques, and 16 negative and 19 positive correlations for feces in ES− (Figure 7A,B). For the ES+ of the no HTN group, the number of positive correlations was very close to that of the negative ones in both saliva and feces, while negative correlations were much more than positive ones in subgingival plaques (Figure 7A,B). In the HTN group, we identified a larger number of positive correlations than negative ones in the oral cavity and a larger number of negative correlations than positive ones in the gut in both ES− and ES+ (Figure 7A,B). For ES−, salivary fungal species in the no HTN group and subgingival fungal species in the HTN group had the most extensive correlations with metabolites, respectively (Figure 7A,B). For ES+, subgingival species had the most extensive correlations with metabolites in both the no HTN group and HTN group. Gut *Cladophialophora bantiana* had the largest number of significant correlations with metabolites among the top 50 fungal species in the no HTN group, and subgingival *Ramularia collo-cygni* had the largest number of significant correlations with metabolites in the HTN group (Figure 7A,B). Together, these results established correlations between plasma metabolites and microbiota at the species level and suggested impairment of interactions between metabolites and microbes in the HTN group.

## 3. Discussion

In this study, we performed 16S rRNA gene sequencing and shotgun metagenomic sequencing of oral/gut microbiota and metabolomics analysis of plasma to investigate associations between microbial composition and host metabolites in participants with or without HTN. Our results identified alterations of plasma metabolites in the HTN group, established associations between altered metabolites and clinical parameters, and revealed the correlations between these metabolites and oral/gut microbes. Simultaneous analysis of bacterial and fungal correlations with plasma metabolites enabled the elucidation of the inter-kingdom influence of microbes on metabolites in participants with or without HTN.

Our results identified alterations of plasma metabolites between the no HTN group and HTN group and established their links with clinical parameters. We identified twenty-nine metabolites in ES− and thirty-four in ES+ significantly enriched in the no HTN group, and three metabolites in ES− and five in ES+ significantly enriched in the HTN group. Of these significantly altered metabolites, 24 in ES− and 27 in ES+ had potential clinical value for discriminating HTN via ROC analysis. Most of the altered plasma metabolites significantly correlated with IL-6, an essential inflammatory mediator with adverse effects on HTN [37]. Inflammation may cause a variety of metabolic alterations in different cells, tissues, and organs [38,39,40]. Interestingly, plasma IL-6 had negative correlations with metabolites enriched in the no HTN group and had positive correlations with metabolites enriched in the HTN group. These results suggested that no HTN-enriched metabolites had protective effects on HTN and HTN-enriched metabolites had adverse influences, and that IL-6 might be an important factor mediating the influence of metabolites on BP.

Our results provide a feasible reference for exploring the influence of microbiota and plasma metabolites on HTN. We showed that the correlation of similarity between salivary bacteria community and plasma metabolome was negative, and that the correlation of similarity between subgingival bacteria community and metabolome was positive. Unlike bacteria, similarity correlations between oral/gut fungal community and metabolome were all negative. These results suggested that the bacterial and fungal kingdoms have a differential association with plasma metabolites. Analyzing the correlations between taxonomic community composition and significantly altered metabolites allowed us to delve deeper into how microbes may affect HTN. Our results showed that the bacterial community composition in saliva, subgingival plaques, and feces all significantly correlated with S1P, and as so with the fungal community composition in feces. S1P has been demonstrated to play important roles in BP regulation [35]. Animal studies have shown both hypotensive and hypertensive effects of S1P [41] because it activates S1P_1_/S1P_3_ receptors in endothelial cells to cause vasorelaxation and S1P_2_/S1P_3_ receptors in vascular smooth muscle cells to cause vasoconstriction [42]. Our data revealed a significant decrease in S1P in HTN and strong correlations between S1P and oral/gut microbiota, suggesting that the associations between S1P and microbiota might be one of the pathways related to microbes and HTN. Mechanistically, how may microbiota influence BP through S1P? Our previous work showed that the S1P-related oral/gut microbiota (e.g., salivary *Streptococcus* and gut *Prevotella*) correlated with erythrocytes and platelets [33]. These results shed some light on this matter, pointing to the influence on the source of S1P by microbiota, given that both erythrocytes and platelets are important sources of plasma S1P [36]. Future studies are warranted to explore the mechanistic insights in more depth.

Our results illustrated strong associations between the oral–gut shared bacteria and significantly altered metabolites. Our results established a close relationship between the oral–gut shared bacteria and plasma metabolites, especially for these bacteria in the gut. Our previous report indicated that the oral–gut transmission of bacteria played a vital role in HTN [33]. These results together suggested that the effect of oral–gut-transmitting bacteria on plasma metabolites might be one of the critical ways they regulated BP. Although the fungi had a larger number of oral–gut shared genera when compared to bacteria, the number of significant associations between these fungi and plasma metabolites were considerably less those between shared bacteria and metabolites. These results highlighted potential differences between bacteria and fungi in influencing plasma metabolites.

Interestingly, the impaired association between bacteria/fungi and plasma metabolites was a striking feature of the participants with HTN. For oral and gut microbiota, the number of positive and negative correlations between bacteria/fungi and metabolites were notably decreased in the HTN group. This reduction of correlations between microbiota and metabolites was most pronounced in gut bacteria and fungi. These alterations of association highlighted the essential role oral/gut microbes might play in HTN. For example, *Bifidobacterium pseudocatenulatum* is a beneficial bacterial species that can play anti-inflammatory roles in diseases such as obesity [43], colitis [44], and liver cirrhosis [45]. In the present results, its diverse associations with plasma metabolites in the no HTN group were completely abolished in the HTN group. These results suggested that a balanced network of microbes and metabolites in normal physiological states may be necessary for maintaining BP.

Taken together, this study identified the differences in plasma metabolites between the no HTN group and HTN group and provided insight into the relationships between human blood metabolome and microbiome interplay in HTN. HTN-specific alterations of the microbiota–metabolites correlation network suggested that the diverse relationships between bacteria/fungi and metabolites might contribute to HTN. Moreover, the strong influences of microbiota and clinical parameters on plasma metabolites provided mechanistical insights into the role of oral/gut microbiota in host metabolism in HTN. Further investigations to identify the functional consequences of the disrupted balance between metabolites and microbiota are warranted for a deeper understanding of the roles of microbiota and metabolites in HTN.

## 4. Materials and Methods

### 4.1. Human Cohort and Sample Collection

A total of 52 participants with HTN and 24 controls without HTN were recruited from the Department of Cardiology at Shanghai Ninth People’s Hospital. The inclusion and exclusion criteria were consistent with our previous works [33,34]. Participants with systolic blood pressure ≥140 mmHg and/or diastolic blood pressure ≥90 mmHg were diagnosed as HTN. Twelve participants were diagnosed with HTN for the first time in this recruitment and did not take the antihypertensive treatment. Participants have more than eight natural teeth. Participants suffering from peripheral artery disease, autoimmune disease, heart failure, renal failure, cancer, irritable bowel syndrome, inflammatory bowel disease, or recurrent aphthous oral ulcers were excluded. Participants were also excluded if they were pregnant or received antibiotic/probiotic treatment or oral/gut surgeries in the previous 2 months.

Participants were required to avoid eating and drinking for at least an hour; then, oral samples were collected after participants rinsed their mouths. Saliva was collected using 50 mL sterile tubes, subgingival plaques were collected using Hu-Friedy subgingival curettes, and feces were freshly collected in stool collection containers. All samples were immediately placed in boxes with ice packs after collection. After 8 h of fasting at least, whole blood samples were collected in tubes with anticoagulants. Blood samples were centrifuged at 3500 rpm for 15 min to obtain plasms. All samples were frozen at −80 °C before use. All samples were run in a single batch for microbiome sequencing and metabolomics detection. All clinical information was collected according to standard clinical protocols. The study was approved by the Institutional Review and Ethics Board of Shanghai Ninth People’s Hospital, Shanghai Jiao Tong University School of Medicine (SH9H-2018-T66-3). Informed consent was signed by all subjects before enrollment.

### 4.2. DNA Extraction

Commercial Kits (M5635-02, Omega Bio-Tek, Norcross, GA, USA) were used to extract genomic DNA according to the manufacturer’s descriptions. A NanoDrop NC2000 spectrophotometer (Thermo Fisher Scientific, Waltham, MA, USA) and agarose gel electrophoresis were used to evaluate the quantity and quality of the extracted DNA. DNA samples were preserved at −20 °C until use.

### 4.3. 16S rRNA Gene Sequencing and Metagenomic Sequencing

The detailed protocol can be found in our previous study [33]. Briefly, 16S rRNA gene libraries were constructed using primers specific to the V3-V4 region and processed using the Illumina MiSeq sequencing platform. Forward and reverse primers were 338F(5′-ACTCCTACGGGAGGCAGCA-3′) and 806R (5′-GGACTACHVGGGTWTCTAAT-3′), respectively. The PCR system included 5 μL of buffer (5×), 0.25 μL of FastPfu DNA Polymerase (5 U/μL), 2 μL (2.5 mM) of dNTPs, 1 μL (10 uM) of each forward and reverse primer, 1 μL of DNA Template, and 14.75 μL of ddH_2_O. PCR products were purified with Vazyme VAHTSTM DNA Clean Beads (Vazyme, Nanjing, China) and quantified using a Quant-iT PicoGreen dsDNA Assay Kit (Invitrogen, Carlsbad, CA, USA) after amplification. Pair-end 2 × 250 bp sequencing of equal amounts of amplicons was performed using the Illumina MiSeq platform with the MiSeq Reagent Kit v3 at Shanghai Personal Biotechnology Co., Ltd. (Shanghai, China). DADA2 was used to filter sequencing reads [46]. Sequence data analyses were performed using QIIME2 and R packages (v3.2.0) [47]. Taxa were annotated by mapping the Greengenes database. The average non-singleton reads per sample were 24,727 for saliva, 21,734 for subgingival plaque, and 37,708 for feces. For taxa annotation of bacteria via 16S rRNA gene sequencing, 29 phyla, 449 genera, and 578 species in saliva were identified; 33 phyla, 364 genera, and 486 species in subgingival plaques were identified; and 32 phyla, 387 genera, and 573 species in feces were identified.

Whole-genome shotgun sequencing was carried out on an Illumina NovaSeq platform at Personal Biotechnology Co., Ltd. Briefly, microbial DNA was processed to construct sequencing libraries with insert sizes of 450 bp using the Illumina TruSeq Nano DNA LT Library Preparation Kit. All libraries were sequenced with the PE150 strategy. After quality control, the reads aligned to the human genome were removed based on Best Match Tagger software. Once quality-filtered reads were obtained, the assembly of reads was executed via MEGAHIT to construct the metagenome for each sample [48], and resulting contigs > 200 bp were preserved for further analysis. A reads-based approach to species annotation of metagenomic sequences via Kraken2 and kaiju was used [49,50]. Taxonomic annotation via alignment with the NCBI RefSeq database was performed by Kraken2 software (https://github.com/DerrickWood/kraken2/wiki, accessed on 12 October 2020). When the sequence annotation rate was less than 30% via Kraken2, taxonomic annotation via mapping with the NCBI NR database was performed by kaiju (https://kaiju.binf.ku.dk/, accessed on 12 October 2020). The average sequencing reads per sample were 285,777,977 for saliva; 81,620,048 for subgingival plaque; and 81,177,495 for feces. For taxa annotation of fungi via metagenomic sequencing, 8 phyla, 182 genera, and 287 species in saliva were identified; 9 phyla, 192 genera, and 302 species in subgingival plaques were identified; 8 phyla, 225 genera, and 371 species in feces were identified.

### 4.4. Untargeted Metabolomics Analysis

Sample pretreatment. All plasma samples were slowly thawed at 4 °C, then 1 mL of pre-cooled methyl alcohol/acetonitrile/water (2:2:1, *v*/*v*) was added to each sample and mixed adequately via vortex. After ultrasonic decomposition in the ice bath for 30 min, the samples were incubated at −20 °C for 10 min to precipitate the protein and then centrifuged at 14,000× *g* at 4 °C for 20 min. The supernatants were collected and dried under vacuum and then stored at −80 °C standby. The samples were re-dissolved in 100 μL of acetonitrile/water (1:1, *v*/*v*), adequately vortexed, and centrifuged (14,000× *g*) at 4 °C for 15 min. The final supernatants were collected for high-throughput liquid chromatography–mass spectrometry/mass spectrometry (LC-MS/MS) analysis.

LC-MS/MS analysis. UHPLC (1290 Infinity LC, Agilent Technologies), HILIC, and RPLC were used to separate the samples. The column temperature was 25 °C and the flow rate was set to 300 μL/min. The composition of the mobile phase of chromatography included buffer A (25 mM ammonium acetate and 25 mM ammonium hydroxide) and buffer B (Acetonitrile). Samples (5 μL) were loaded and placed in an auto-sampler at 4 °C, and then analyzed randomly to minimize deviations caused by fluctuations in the detection signals. Detections were performed in both the positive ion mode and negative ion mode. UHPLC coupled to a quadrupole time-of-flight (AB SCIEX TripleTOF 6600) was used for the analyses.

Data processing. ProteoWizard MS Convert was used to convert the raw MS data (wiff.scan files) into MzXML files. XCMS was used for feature detection, retention time correction, and alignment. The metabolites were identified using mass accuracy (<25 ppm) and multi-dimensional matching MS/MS data with public databases of Bio cyc, HMDB, METLIN, HFDB, and LIPID MAPS as well as the database of standards generated by Personal Biotechnology Co., Ltd.

Metabolites with more than 50% missing values were removed before the following analysis. The total peak area of the metabolic data was normalized, and the data were performed with Pareto-scaling processing in SIMCA-P software. Based on LC-MS/MS analysis, 2893 and 2950 chromatographic peaks were identified in the positive ion mode and negative ion mode, respectively. Ten superclasses covering a wide range of biochemicals including lipids, hormones and transmitters, peptides, carbohydrates, organic acids, vitamins and cofactors, and steroids were identified by blasting our untargeted metabolomics results in the Human Metabolome Database (HMDB).

## 5. Statistical Analysis

Data were analyzed in RStudio73 with R 4.2.1. SPSS was used to construct the operating characteristic and precision–recall curves and calculate the area under curve. A supervised model of PLS-DA was used to assess the alterations of metabolites among groups and to calculate the variable importance for the projection. To construct the PLS-DA model, plasma samples were divided into a training set and a testing set using the sample set partitioning through the algorithm of the joint x–y distance. PLS-DA was performed using SIMCA-P software to cluster the sample plots. The significantly altered metabolites in plasma metabolites were determined via Student’s *t*-test. Spearman’s correlations among microbiota, clinic parameters, and metabolites were calculated and visualized using the corrplot R software package. Adjusted *p*-values (false discovery rate, FDR) for multiple comparisons were analyzed using Benjamini–Hochberg tests throughout the analysis. The partial Mantel correlations between microbial composition and plasma metabolites were tested using the linkET R software package. Correlation networks between taxa and metabolites were built using Gephi 0.9.2.

## Figures and Tables

**Table 1:** Demographics of enrolled participants.

Characteristics	No HTN	HTN	*p* Values (No HTN vs. HTN)
Sex	15 women; 9 men	30 women; 22 men	0.69
Age (years)	66.29 ± 1.42	69.21 ± 0.69	0.073
Height (cm)	163.40 ± 1.99	164.10 ± 1.02	0.77
Weight (kg)	64.93 ± 2.16	65.35 ± 1.37	0.87
BMI (kg/m^2^;)	24.56 ± 0.82	24.33 ± 0.44	0.79
SBP (mmHg)	120.6 ± 3.31	133.10 ± 2.01	0.0025
DBP (mmHg)	76.58 ± 1.27	79.10 ± 1.23	0.16
Drinkers, n (%)	1 (4.17%)	2 (3.84%)	0.31
Diabetes mellitus, n (%)	0 (0.00%)	6 (11.54%)	NA
Antihypertensive treatment, n (%)	0 (0.00%)	40 (76.92%)	NA
NaÏve to antihypertensive treatment, n (%)	NA	12 (23.08%)	NA

Data are shown as mean ± SEM or n (%). HTN, hypertension; BMI, body mass index; SBP, systolic blood pressure; DBP, diastolic blood pressure; NA, not applicable. Pearson’s chi-squared test was performed for statistical analyses of sex and drinkers between no HTN and HTN. Student’s *t*-test was performed for statistical analyses of age, height, weight, BMI, and blood pressure between no HTN and HTN.

## Data Availability

Raw sequences of microbiota were uploaded to the Sequence Read Archive of NIH with accession numbers PRJNA764503, PRJNA765566, and PRJNA774166. Please contact the corresponding author to acquire original metabolomics data.

## Supplementary Materials

The following supporting information can be downloaded at: https://www.mdpi.com/article/10.3390/nu15092074/s1. Table S1: Area Under Curve of ES (-); Table S2: Area Under Curve of ES (+); Table S3: Chi-squared test; Figure S1: PCA plots of plasma metabolites between no HTN and HTN; Figure S2: ROC curve of plasma metabolites for classification of no HTN and HTN.
