# Supplementary material for: Integrated Omic Analysis of Human Plasma Metabolites and Microbiota in a Hypertension Cohort
Source: Nutrients. 2023 Apr 25;15(9):2074. doi: 10.3390/nu15092074 (PMC10180880; doi:10.3390/nu15092074)
Supplement: Supplementary file 1 [file nutrients-15-02074-s001.zip › nutrients-2307808-supplementary.pdf]

Supplemental tables

Table S1 Area Under Curve of ES (-)

Metabolite ID	Metabolite name	Area	Standard error[a]	Asymptotic Significance[b]	Asymptotic 95%confidence interval	
					Lower Limit	Upper Limit
M0320	epi-jasmonic acid	0.681	0.069	0.012	0.545	0.817
M0147	Maltitol	0.646	0.070	0.042	0.508	0.784
M0099	Chlorphentermine	0.716	0.062	0.003	0.595	0.838
M0290	Crotonic acid	0.669	0.069	0.018	0.534	0.804
M0144	2-Deoxyhexopyranose	0.654	0.067	0.032	0.523	0.784
M0376	(E)-4-Methoxycinnamic acid	0.696	0.065	0.006	0.567	0.824
M0321	guaietolin	0.673	0.068	0.016	0.539	0.807
M0075	(E)-3,4,5-Trimethoxycinnamic acid	0.663	0.069	0.023	0.528	0.799
M0271	LU3453000	0.683	0.069	0.010	0.549	0.818
M0088	3-Hydroxy-3-(2,3,4-trimethoxyphenyl)propanoic acid	0.670	0.069	0.018	0.535	0.804
M0021	DL-Lactic Acid	0.631	0.071	0.069	0.491	0.770
M0259	(2S)-2-Amino-8-hydroxyoctanoic acid	0.708	0.062	0.004	0.585	0.830
M0282	S-3-oxodecanoyl cysteamine	0.731	0.062	0.001	0.610	0.852
M0253	Guvacine	0.675	0.073	0.015	0.531	0.818
M0233	2-Amino-3-hydroxyoctadecyl dihydrogen phosphate	0.645	0.069	0.043	0.510	0.780
M0062	Sphingosine 1-phosphate	0.647	0.071	0.041	0.508	0.785
M0221	Lauramide	0.680	0.068	0.012	0.546	0.814
M0268	4-Methylcarbostyril	0.651	0.069	0.036	0.515	0.787
M0310	Umbelliferone	0.655	0.069	0.031	0.519	0.790
M0155	1-O-	0.640	0.071	0.051	0.502	0.779

	Arsonopentofuranose					
M0058	2,4-Dichlorotoluene	0.631	0.067	0.069	0.499	0.763
M0222	N-dodecanoylsphinganine	0.696	0.069	0.006	0.561	0.830
M0029	methadone-d9	0.659	0.072	0.026	0.518	0.801
M0063	DL-Arginine	0.675	0.068	0.014	0.542	0.809

Table S2 Area Under Curve of ES (+)

Metabolite ID	Metabolite name	Area	Standard error[a]	Asymptotic Significance[b]	Asymptotic 95%confidence interval	
					Lower Limit	Upper Limit
M0386	4-Coumaric acid	0.655	0.074	0.031	0.509	0.801
M0347	epi-jasmonic acid	0.681	0.069	0.012	0.545	0.817
M0192	2-Deoxyhexopyranose	0.679	0.071	0.013	0.539	0.818
M0128	Hexitol	0.655	0.073	0.031	0.511	0.799
M0200	Maltitol	0.645	0.070	0.043	0.507	0.783
M0186	Hydroxytriazolam	0.708	0.069	0.004	0.572	0.843
M0124	Chlorphentermine	0.716	0.062	0.003	0.595	0.838
M0247	(2Z)-3-{5-[(4Z)-5-(Methylsulfanyl)-4-penten-2-yn-1-yl]-2-furyl}acrylaldehyde	0.632	0.070	0.065	0.494	0.770
M0312	D-(+)-Maltose	0.628	0.071	0.074	0.489	0.767
M0348	guaietolin	0.673	0.068	0.016	0.539	0.807
M0091	3-(6,7-Dimethoxy-1,3-benzodioxol-5-yl)-2-propen-1-ol	0.663	0.069	0.023	0.528	0.799
M0105	P-Gal	0.670	0.069	0.018	0.535	0.804
M0307	S-3-oxodecanoyl cysteamine	0.731	0.062	0.001	0.610	0.852
M0289	(2S)-2-Amino-4-hexynoic acid	0.670	0.073	0.018	0.527	0.813
M0240	1-[(1Z,9Z)-octadecadienyl]-sn-glycero-3-phosphocholine	0.659	0.075	0.027	0.512	0.805
M0261	2-Amino-3-hydroxyoctadecyl dihydrogen phosphate	0.645	0.069	0.043	0.510	0.780
M0066	D-Erythro-sphingosine 1-phosphate	0.649	0.071	0.038	0.511	0.787
M0250	Lauramide	0.680	0.068	0.012	0.546	0.814
M0003	1-[(11Z)-octadecenoyl]-sn-	0.657	0.074	0.029	0.512	0.802

	glycero-3-phosphocholine					
M0298	4-Methylcarbostyril	0.651	0.069	0.036	0.515	0.787
M0338	4-Hydroxycoumarin	0.655	0.069	0.031	0.519	0.790
M0180	1-O-Arsonopentofuranose	0.640	0.071	0.051	0.502	0.779
M0067	2,4-Dichlorotoluene	0.631	0.067	0.069	0.499	0.763
M0106	4-Hydroxycyclophosphamide	0.713	0.063	0.003	0.590	0.837
M0251	N-dodecanoylsphinganine	0.696	0.069	0.006	0.561	0.830
M0160	Benzothiazole	0.742	0.058	0.001	0.628	0.856
M0069	DL-Arginine	0.675	0.068	0.014	0.542	0.809
M0205	methyl isoquinoline-3-carboxylate	0.671	0.064	0.017	0.545	0.797

Table S3 Chi-squared test

	Value	df	Asymptotic Significance （2-sided）	Exact Sig. (2-sided)	Exact Sig. (1-sided)
Pearson chi-squared	14.256[a]	1	0.000160		
Continuity Correction[b]	13.610	1	0.000225		
Likelihood Ratio	14.350	1	0.000152		
Fisher's Exact Test				0.000188	0.000108
Linear-by-Linear Association	14.237	1	0.000161		
N of Valid Cases	737				

a. 0 cells (0.0%) have expected count less than 5. The minimum expected count is 86.19.

b. Computed only for a 2x2 table.

Supplemental figures

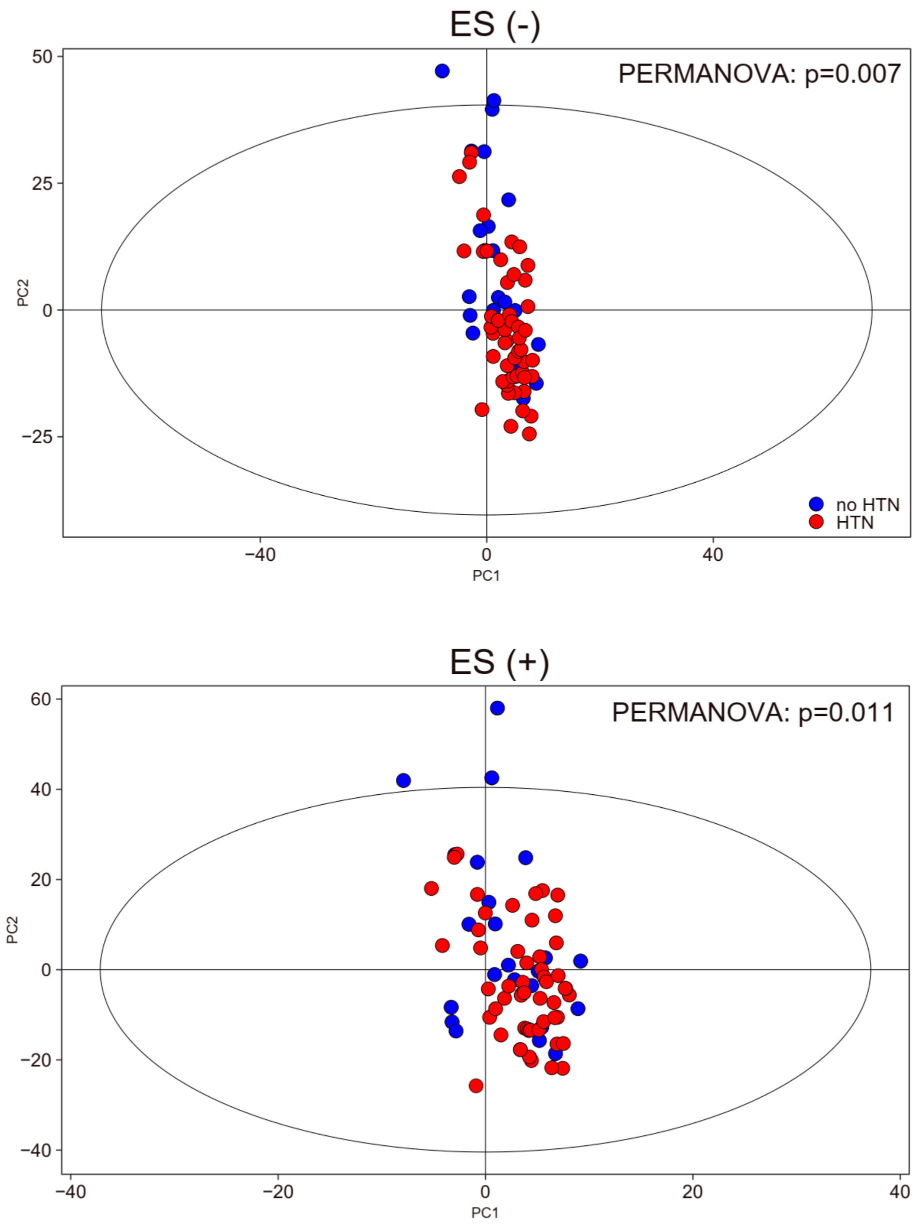

Fig. S1 PCA plots of plasma metabolites between no HTN and HTN. PERMANOVA (permutational analysis of variance) test was used to evaluate the overall metabolite composition between no HTN and HTN in ES (-) and ES (+). PCA: Principal component analysis. n=24:36 (no HTN vs HTN).

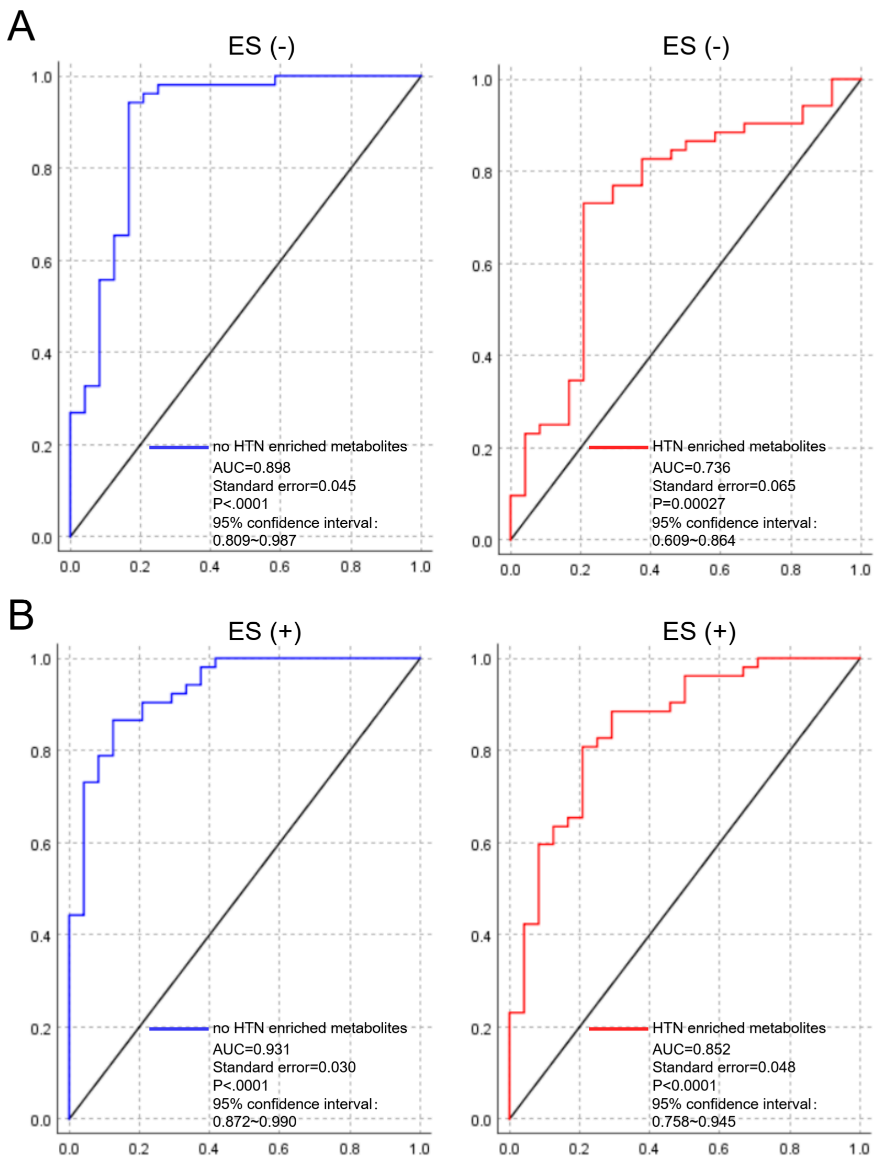

Fig. S2 ROC curve of plasma metabolites for classification of no HTN and HTN. A-B Metabolites enriched in no HTN and HTN were combined to construct the ROC curve in ES (-) and ES (+), respectively. n=24:36 (no HTN vs HTN).
